# Cost of treating inpatient falciparum malaria on the Thai-Myanmar border

**DOI:** 10.1186/1475-2875-13-416

**Published:** 2014-10-29

**Authors:** Shwe Sin Kyaw, Tom Drake, Ronatrai Ruangveerayuth, Wirongrong Chierakul, Nicholas J White, Paul N Newton, Yoel Lubell

**Affiliations:** Mahidol Oxford Tropical Medicine Research Unit, Faculty of Tropical Medicine, Mahidol University, Bangkok, Thailand; Centre for tropical Medicine, Nuffield Department of Medicine, University of Oxford, Oxford, UK; Mae Sot Hospital, Mae Sot, Tak, Thailand

**Keywords:** Artesunate, Quinine, Cost, Severe malaria, Malaria

## Abstract

**Background:**

Despite demonstrated benefits and World Health Organization (WHO) endorsement, parenteral artesunate is the recommended treatment for patients with severe *Plasmodium falciparum* malaria in only one fifth of endemic countries. One possible reason for this slow uptake is that a treatment course of parenteral artesunate is costlier than quinine and might, therefore, pose a substantial economic burden to health care systems. This analysis presents a detailed account of the resources used in treating falciparum malaria by either parenteral artesunate or quinine in a hospital on the Thai-Myanmar border.

**Methods:**

The analysis used data from four studies, with random allocation of inpatients with falciparum malaria to treatment with parenteral artesunate or quinine, conducted in Mae Sot Hospital, Thailand from 1995 to 2001. Detailed resource use data were collected during admission and unit costs from the 2008 hospital price list were applied to these. Total admission costs were broken down into five categories: 1) medication; 2) intravenous fluids; 3) disposables; 4) laboratory tests; and 5) services.

**Results:**

While the medication costs were higher for patients treated with artesunate, total admission costs were similar in those treated with quinine, US$ 243 (95% CI: 167.5-349.7) and in those treated with artesunate US$ 190 (95% CI: 131.0-263.2) (P = 0.375). For cases classified as severe malaria (59%), the total cost of admission was US$ 298 (95% CI: 203.6-438.7) in the quinine group as compared with US$ 284 (95% CI: 181.3-407) in the artesunate group (P = 0.869).

**Conclusion:**

This analysis finds no evidence for a difference in total admission costs for malaria inpatients treated with artesunate as compared with quinine. Assuming this is generalizable to other settings, the higher cost of a course of artesunate should not be considered a barrier for its implementation in the treatment of malaria.

## Background

Although falciparum malaria is an entirely preventable and treatable disease, it caused an estimated 627,000 deaths in 2012 [1]. Malaria also has a devastating economic [2] and social impact [3], being both a root cause and consequence of poverty, contributing to the vicious cycle of poverty and ill health. In endemic countries malaria is a common cause of inpatient admissions and as access to health care improves with economic development, a larger proportion of patients with severe malaria are likely to be admitted in time to receive potentially life-saving treatment. Providing the most efficacious treatment to these patients will improve survival.

Parenteral artesunate has been shown to be considerably superior to quinine in the management of severe malaria, with a reduction in mortality from 10.9% to 8.5% in Africa [4] and from 22% to 15% in Asia [5]. Subsequent cost-effectiveness analyses confirmed the economic superiority of artesunate over quinine in the management of malaria in both the African [6] and Asian [7] settings, with an incremental cost per death averted of approximately $150 in both settings, and is therefore highly cost-effective.

Despite these demonstrated benefits and WHO endorsements, parenteral artesunate is the recommended first-line treatment for patients with severe malaria in a small number of endemic and non-endemic countries, including only two countries in sub-Saharan Africa [1]. One possible reason for this slow uptake is the prima facie notion that artesunate (US$ 2.3 per vial) is costlier than quinine (US$ 0.2 per vial) [8] and would therefore pose a substantial economic burden to healthcare systems. While previous economic evaluations [6, 7] found the costs of admission for the two regimens were comparable, but they did not benefit from detailed data on the resources used to manage malaria admissions. This study estimates and compares the costs of treating adult malaria inpatients in a hospital on the Thai-Myanmar border with either parenteral artesunate or quinine, building on a detailed account of the resources used in caring for these patients.

## Methods

### Study site

This analysis used data from four studies conducted in Mae Sot Hospital in Thailand from 1995 to 2001. The hospital is located in Tak Province near the Thai-Myanmar border and serves a predominantly rural population of Thai farmers and Burmese and Karen displaced persons. Malaria transmission in the area is seasonal and of low intensity [9]. At the time of studies, 30% of the Thai population (18 million people) mostly from low socioeconomic groups as well as the migrant population on the Thai Myanmar border had no health insurance and no assured access to free medical care, although exemptions were granted by hospitals on a case-by-case basis. Out-of-pocket payment accounted for 33% of total health expenditure in 2001[10].

Mae Sot Hospital has 420 beds and facilities for haematology, biochemistry, microbiology, radiology, ventilation, central line insertion, haemodialysis, and peritoneal dialysis. Arterial blood gas and blood pH determination of haemofiltration were not available.

Detailed resource use data were collected on specifically designed forms in four studies in which patients were randomized to artesunate or quinine treatment. In total, resource use data were available for 100 patients. These consisted of 42 patients recruited in a randomized control trial on the treatment of severe falciparum malaria by either parenteral artesunate or quinine [11]; 34 patients recruited in a randomised trial of artesunate alone versus combined artesunate and quinine (only those in the artesunate arm were included in the current study) [12]; 19 patients recruited to a study of red cell survival [13]; and five patients recruited to a study of retinal findings in malaria [14]. Ethical clearance for all these studies was approved by the Ministry of Public Health, Government of Thailand.

### Cost estimation

This analysis estimates patient-level direct medical costs of managing inpatient falciparum malaria, calculated by multiplying the quantities of resources used by their unit costs (the ‘ingredients’ approach). Direct medical costs included management of care, the costs of services, supplies and equipment. Patients’ notes were reviewed and a list made of all medications, fluids, equipment, laboratory tests, and services provided. The hospital bills for each patient were also entered into a database. If a bill listed more resources than those recorded on the data collection forms, the latter were used to determine the quantities of resources used. Unit costs were taken from the 2008 hospital price list as an approximation of the actual economic cost.

Total admission costs were broken down into five categories: 1) medication; 2) intravenous fluids; 3) disposables; 4) laboratory tests; and, 5) services. Medication included the costs of all drugs used during admission including anti-malarials, antibiotics and supportive treatment. Intravenous fluid costs included all fluids used during admission. Disposable equipment costs were those of syringes, needles, cannulas, infusion sets, etc. All investigations such as full blood count, serum biochemistry, chest X-ray, and ECG were included in the laboratory costs. Services included activities such as administration of fluids and medications, as well as the overhead cost per admission day in either the general wards or the ICU.

Other nurses and physicians activities that were not amenable for inclusion in the costing but could affect the relative time spent attending to patients in each treatment group are summarised independently. These consist of the number of times blood pressure, temperature, pulse rates, and respiratory rates were measured, and intake/output recordings. The number of pages in patient notes written by nurses and physicians was also recorded as an indication of nurse and physician time spent attending to the patients.

All unit costs were obtained at 2008 prices in Thai Baht (THB) and adjusted for inflation using a consumer price index, and then converted to US dollars using the 2012 exchange rate (US$1 = 31 THB).

### Statistical analysis

Patient-level data were entered in a Microsoft Excel spreadsheet. All analyses were performed using R statistical software, version 3.1 (Vienna, Austria). Costs for each treatment group were summarized and stratified by severity. Independent sample T-test was performed for hypothesis testing of continuous variables. To deal with skewed cost data and to reduce the effect of random sampling error, non-parametric bootstrapping simulation was carried out to select 1000 random samples with replacement from the set of total admission costs in each treatment group.

## Results

Of the 100 patients for whom data were available, the majority of patients were male (75%) and the mean age was 32 years (IQR: 22–41 years). Fifty-seven percent of the patients originated from Myanmar. Karen and Burmese ethnic groups were most common, at 28 and 27%, respectively. Almost all patients of Myanmar origin worked as general labourers. Among the 100 admissions, 59 were classified as severe malaria [15] and 41 had uncomplicated malaria but required parenteral therapy because of hyperparasitaemia or inability to take oral medication.

Of all admitted malaria patients, 29 were treated with parenteral quinine and 71 by parenteral artesunate (the imbalanced distribution is due to inclusion of 34 patients treated with artesunate alone but not those in the artesunate + quinine arm in the same study [12]). The overall mortality rate amongst all patients was 8% (95% CI 3-13%). In the artesunate group 4% (95% CI 0-7%) died as compared with 17% (95% CI 3-30%) of patients receiving quinine. Length of hospital stay was different in the two groups, with a mean of seven days (95% CI 5.3-9.3) in the quinine group as compared with four days (95% CI 3.4-4.6) for artesunate. This difference was apparent for patients with uncomplicated malaria treated with quinine as compared with artesunate (4.1 *vs* 2.7 days; P = <0.001) but was not significantly different in severe malaria patients (6.8 *vs* 5.4 days; P = 0.37). In patients who died, the mean time from the start of anti-malarial treatment to death was 70 hours; this was shorter in quinine-treated patients at 27 hours (95% CI 12–45) than artesunate-treated patients at 140 hours (95% CI 31–323).

While the cost of medication is lower in patients receiving quinine, all other costs were considerably higher in quinine-treated patients. The mean total admission cost per patient treated with quinine was almost 30% higher than for patients treated with artesunate, but this was not statistically significant (P = 0.37). Table 1 presents the individual component costs and full admission costs for patients treated with artesunate or quinine, in total and stratified by severity.

**Figure 1:**
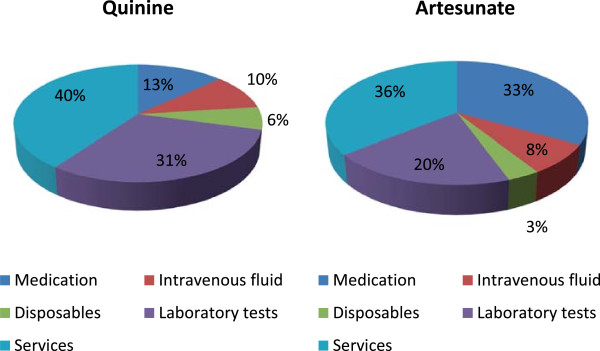
**Cost components of treatment of inpatient falciparum malaria with quinine and artesunate.**

For patients with severe malaria the mean total admission cost was similar in the two arms. In the quinine group, the mean total admission cost per patient was $298 (95% CI: 203–438) whilst for artesunate patients it was US$284 (95% CI: 281–407) (P = 0.869). Figures 1 shows the proportional contribution of each component to total admission costs. The main cost component was services, accounting for almost 40% of total admission costs.

**Table 1:** **Mean admission cost in US dollars in the treatment of falciparum malaria (2012)**

Components	Artesunate	Quinine	P-value
**All malaria patients (n = 100)**			
Medication	63.1 (95% CI: 38.1-96.2)	32.1 (95% CI: 18.6-51.8)	0.077
Intravenous fluid	15.0 (95% CI: 10.7-19.6)	24.0 (95% CI: 17.1-32.1)	0.046
Disposables	6.6 (95% CI: 3.8-9.8)	15.2 (95% CI: 8.5-23.8)	0.015
Laboratory tests	37.5 (95% CI: 26.5-50.2)	74.6 (95% CI: 49.8-107.6)	0.008
Services	68.1 (95% CI: 47.6-92.4)	97.3 (95% CI: 61.8-146.4)	0.258
Total costs	190.3 (95% CI: 131.0-263.2)	243.1 (95% CI: 167.5-349.7)	0.375
**Uncomplicated malaria (n = 41)**			
Medication	31.6 (95% CI 29.4-33.8)	16.5 (95% CI: 8.2-26.1)	<0.001
Intravenous fluid	4.5 (95% CI: 3.7-5.4)	10.1 (95% CI: 7.6-13.3)	0.004
Disposables	1.5 (95% CI: 1.2-1.8)	2.9 (95% CI: 1.8-4.2)	0.002
Laboratory tests	14.2 (95% CI: 10.3-17.9)	24.6 (95% CI: 14.2-37.3)	0.125
Services	30.7 (95% CI: 23.9-30.0)	44.4 (95% CI: 31.4-56.1)	0.088
Total costs	82.5 (95% CI: 72.3-93.7)	98.5 (95% CI: 68.6-129.6)	0.353
**Severe malaria (n = 59)**			
Medication	90.5 (95% CI: 44.4-148.6)	38.0 (95% CI: 20.4-65.3)	0.085
Intravenous fluid	24.0 (95% CI: 16.9-32.0)	29.3 (95% CI: 20.3-39.7)	0.412
Disposables	11.0 (95% CI: 6.1-16.5)	19.8 (95% CI: 11.4-30.3)	0.126
Laboratory tests	57.8 (95% CI: 39.9-80.1)	93.7 (95% CI: 62.3-139.3)	0.115
Services	100.6 (95% CI: 66.5-141)	117.4 (95% CI: 71.9-180.9)	0.636
Total costs	283.8 (95% CI: 181.3-407)	298.2 (95% CI: 203.6-438.7)	0.869

In addition to the activities included in services cost category, patients receiving quinine required considerably more nursing and physician time, as indicated by 1.5 to 3 times as many nursing monitoring activities. The time spent by physicians attending to patients treated with quinine appeared to be higher as indicated by the larger number of pages in their records. These differences are explained by the longer duration of admission and were not evident when compared on a per-day basis. Table 2 shows the breakdown in monitoring activities between the two arms.

**Table 2:** **Mean number of monitoring activities per patient receiving artesunate or quinine**

Monitoring activities	Artesunate	Quinine	P-value
Blood pressure	13	23	0.02
Pulse rate and temperature	20	31	0.08
Respiratory rate	21	31	0.09
Number of fluid intake/output recordings	3	5	0.06
Number of pages of notes recorded by nurses	4	6	0.14
Number of pages of notes recorded by physicians	3	6	0.002

## Discussion

There are numerous reasons that could explain the slow uptake of artesunate in national treatment guidelines and routine use, including the need for re-training and establishing new procurement and distribution systems, as well as clinician resistance to change. Given the untapped potential gains offered by artesunate these all require urgent attention. Here simply focus on the perception that malaria admissions treated with artesunate might be costlier as a barrier to implementation. While this study confirms the higher cost of medication in patients treated with artesunate, the study finds no evidence of higher total admission costs, with some indication of cost savings for patients treated with artesunate. This can be explained to a great extent by the shorter time to discharge and associated service costs as well as the lower quantity of disposables and fluids required for administering artesunate.

The estimated admission costs of inpatients with malaria in this study are higher than those calculated in previous studies [6, 7]. In addition to the more detailed methods used here that could capture a broader range of inputs, the differences might be explained by the use of hospital price list as unit costs, which could be higher than their economic value. Despite total costs per admission for both treatment groups being higher in this study, the difference between the treatment groups is non-significant, with some indication of lower costs for patients treated with artesunate. The incremental cost per DALY averted, therefore, will remain very low, as indicated in other studies, or potentially negative, implying that treatment with artesunate dominates quinine being both less costly and more effective.

### Limitations

There are important limitations to this study. While it benefits from detailed records of the resources used in the two treatment arms, the unit costs applied to these quantities were based on the hospital price list. A cost/charge ratio was not available for this setting to adjust prices to reflect their economic value. This will not however affect the conclusion regarding the relative costs of the two treatments, as long as the variation in unit costs for the different items in other settings is largely proportional.

Another limitation is that the resource use data were collected around the late 1990s and in the context of clinical trials, therefore, their management might not be entirely reflective of routine care in other settings. This limitation is likely outweighed by the benefit of randomization and head to head comparison of the two treatments. Typically health systems do not implement multiple first-line therapies and direct cost comparisons cannot easily be made.

In terms of the statistical analysis the patients originated from four different studies and ideally the analysis would be stratified or controlled for accordingly. The sample size however was too small to allow for this and while the studies were different, the patients were cared for by the same clinical team. The analysis of differences in length of hospital stay between the two groups was limited and did not account for competing risks associated with differences in mortality. A more effective treatment can in fact be ‘penalized’ with longer length of stay in surviving patients (who would otherwise have died early in their admission). More advanced statistical analyses than those performed here can attempt to tease these interacting factors apart, but given the evidence regarding clinical superiority of artesunate along with its cost-neutral or cost-saving implications this would have limited added value.

## Conclusion

This study finds no evidence for a difference in total economic cost of care between inpatients treated with quinine and those treated with artesunate, despite substantial differences in drug prices. This analysis has certain limitations including small sample size, the age of the data used and the use of hospital price list as a proxy for health system costs. Nevertheless, the global uptake (or lack thereof) is of major importance in global health. This study, with its limitations, suggests that financial considerations should not prevent the switch from quinine to artesunate for the treatment of malaria admissions.
